# The role of photosynthesis and amino acid metabolism in the energy status during seed development

**DOI:** 10.3389/fpls.2014.00447

**Published:** 2014-09-03

**Authors:** Gad Galili, Tamar Avin-Wittenberg, Ruthie Angelovici, Alisdair R. Fernie

**Affiliations:** ^1^Department of Plant Sciences, The Weizmann Institute of ScienceRehovot, Israel; ^2^Max-Planck-Institut für Molekulare PflanzenphysiologiePotsdam-Golm, Germany; ^3^Department of Biochemistry and Molecular Biology, Michigan State UniversityEast Lansing, MI, USA

**Keywords:** seed development, metabolism and bioenergetics, photosynthesis, branched chain amino acids, TCA cycle

## Abstract

Seeds are the major organs responsible for the evolutionary upkeep of angiosperm plants. Seeds accumulate significant amounts of storage compounds used as nutrients and energy reserves during the initial stages of seed germination. The accumulation of storage compounds requires significant amounts of energy, the generation of which can be limited due to reduced penetration of oxygen and light particularly into the inner parts of seeds. In this review, we discuss the adjustment of seed metabolism to limited energy production resulting from the suboptimal penetration of oxygen into the seed tissues. We also discuss the role of photosynthesis during seed development and its contribution to the energy status of developing seeds. Finally, we describe the contribution of amino acid metabolism to the seed energy status, focusing on the Asp-family pathway that leads to the synthesis and catabolism of Lys, Thr, Met, and Ile.

## INTRODUCTION

Seeds are the major organ responsible for the evolutionary upkeep of the plant lineage. They store the genetic material of the plant within the embryo, thus guarantying continuation of the plant’s life cycle in the next generation. Seed development is highly regulated, with distinct transcript, protein and metabolite switches occurring in a concerted manner throughout its progression (Fait et al., 2006; Sreenivasulu et al., 2008; Xu et al., 2008; Angelovici et al., 2010). Seeds produce a great variety of storage compounds, among them a myriad of carbohydrates (especially starch), storage proteins, and storage lipids. These provide – both directly in the form of food and indirectly in the form of feed ∼70% of the world’s human caloric intake (Sreenivasulu and Wobus, 2013). Storage metabolites are subsequently used as nutrient and energy sources to support early stages of seed germination, while the seedling is still heterotrophic. Production of storage compounds whose generation by respiration becomes limiting due to a limitation of oxygen penetration into the dense seed tissues requires a considerable amount of energy. The requirement for oxygen and hence energy production becomes particularly critical during reserve metabolites accumulation and seed desiccation, in which the dense seed tissues limit the oxygen penetration and thus limit mitochondrial energy production (Weber et al., 2005; Angelovici et al., 2010). Photosynthesis in green seeds, and other energy conservation mechanisms, such as a decrease of overall respiration rates during embryo development, somewhat relieve the major drop in energy demand, but do not sufficiently compensate for the energy requirement (Weber et al., 2005; Angelovici et al., 2010). In seeds of the model plant *Arabidopsis thaliana*, amino acid metabolism is modulated so that many amino acids are synthesized and accumulated during seed desiccation (Fait et al., 2006). In recent years, several lines of evidence have implied that amino acids are not only used for the synthesis of storage proteins, but also, upon energy demand, can be catabolized and their catabolic products fed into the TCA cycle to generate energy (Zhu and Galili, 2004; Angelovici et al., 2010; Galili, 2011). This was also shown for plants exposed to extended darkness, another condition characterized by energy deprivation (Ishizaki et al., 2005, 2006; Araujo et al., 2010). In this mini-review, we describe lines of evidence demonstrating the contribution of photosynthesis and amino acid metabolism to the energy production in developing seeds and the contribution of amino acid catabolism to the energy status of seedlings exposed to extended darkness.

## DEVELOPING SEEDS FACE AN EXTREME SHORTAGE OF ENERGY DUE TO LIMITED PENETRATION OF OXYGEN, PARTICULARLY INTO THE INNER SEED PARTS

**FIGURE 1:**
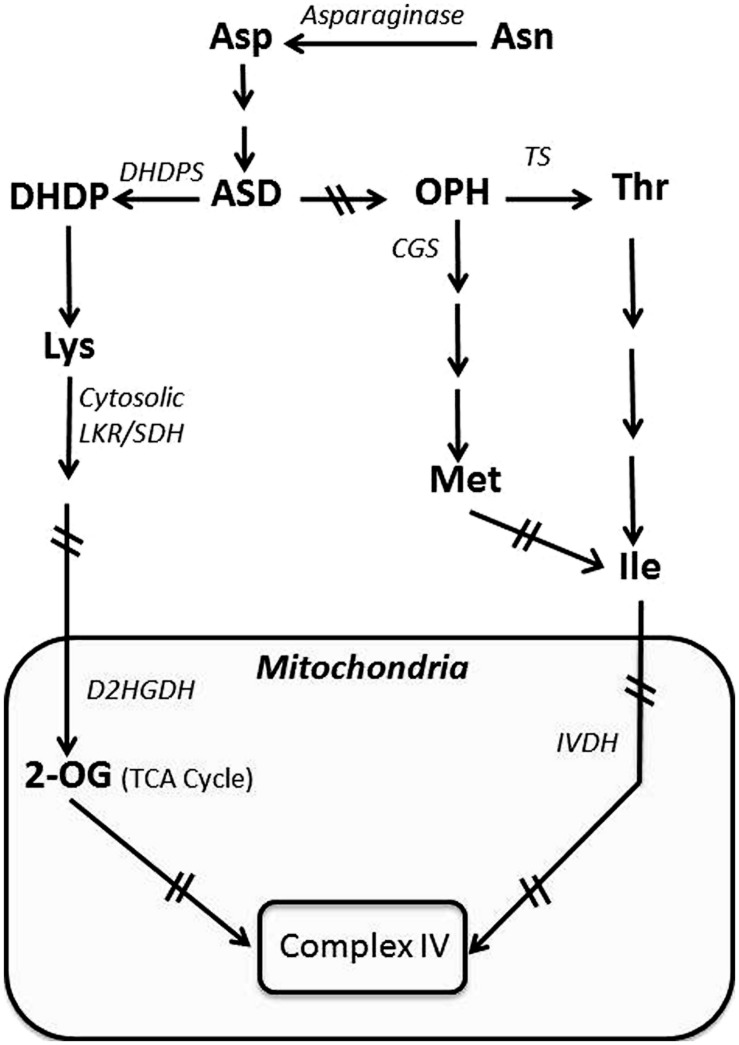
**Schematic diagram showing the synthesis of the amino acids of the Asp-family pathway and their catabolism by the TCA cycle.** The mitochondrial enzymatic complex is highlighted by a black box. Abbreviations: 2-OG, 2-oxoglutarate; ASD, aspartic semialdehyde dehydrogenase; CGS, cystathionine gamma synthase; D2HGDH, 2-D-hydroxyglutarate dehydrogenase; DHDP, dihydrodipicolinate; DHDPS, dihydrodipicolinate synthase; IVDH, isovaleryl-CoA dehydrogenase; LKR/SDH, lysine ketoglutarate reductase/saccharopine dehydrogenase; OPH, *O*- phospho_L_homoserine; TS, Thr synthase. Arrows with two crossed lines represent several enzymatic reactions.

Since developing seeds are quite dense, they suffer from limited penetration of oxygen, particularly into the inner seed tissues. The shortage of oxygen extensively limits energy-requiring metabolic processes crucial for seed development and embryo maturation (Vigeolas et al., 2003, 2011; van Dongen et al., 2004). In developing seeds of dicotyledonous (dicot) plant species, photosynthesis in the developing embryo contributes a considerable amount of oxygen to the seed tissue, which further fuels energy-generating biochemical pathways, such as glycolysis and respiration (Rolletschek et al., 2003; Ruuska et al., 2004; Borisjuk et al., 2005; Tschiersch et al., 2011, 2012). This is in contrast to developing non-green seeds of monocotyledonous (monocot) plants that do not generate energy via photosynthesis, and hence must depend on alternative energy-generating metabolic processes in addition to glycolysis and respiration in order to fuel seed development. The chemical donors for energy-generation are hypothetically produced in the vegetative parts of the plant, using particularly energy derived from photosynthesis, and are transported to the developing seeds (van Dongen et al., 2004). A number of different metabolites produced in the vegetative tissues are transported to the seeds; however, at a quantitative level, the amino acid Asn, which accumulates to relatively high levels during senescence, is particularly notable as a major energy source (Credali et al., 2013). Its role as an energy donor occurs via the conversion of Asn into Asp, the direct precursor of the branched Asp-family pathway (**Figure 1**). The Asp-family pathway synthesizes the amino acids Lys, Thr, Met, and Ile, which, under conditions of energy shortage, are further catabolized by the TCA cycle to generate energy (Galili, 2011). An energy shortage does not occur only in seeds, but also in vegetative tissues that lack photosynthesis, and upon exposure of plants to extended darkness. Under these energy shortage conditions, Lys is directly catabolized into the TCA cycle, while Thr and Met are converted into Ile, which is then directly catabolized into the TCA cycle (Joshi et al., 2006; Araujo et al., 2010). We focus in this review on the catabolism of Lys and Ile in respect to the contribution of amino acid catabolism to the energy status either during seed development or upon exposure to extended darkness.

## CONTRIBUTION OF SEED PHOTOSYNTHESIS TO OXYGEN AVAILABILITY IN DEVELOPING SEEDS

A significant amount of information on the role of seed photosynthesis for the synthesis of seed reserve compounds has been gathered at the IPK Institute for Plant Genetics in Gatersleben Germany (), where they analyzed developing soybean seeds, which possess active seed photosynthesis (Rolletschek et al., 2003; Ruuska et al., 2004; Borisjuk et al., 2005; Tschiersch et al., 2011, 2012). Light intensity and oxygen level progressively decrease with a progressive increase in seed depth (Vigeolas et al., 2003). Oxygen level is highest in light illuminated seeds, progressively lower in seeds that are not illuminated and lowest in seeds exposed to darkness. The ATP/ADP ratio is markedly higher in developing seeds exposed to light, indicating that photosynthesis in illuminated seeds contributes to the production of energy (Borisjuk et al., 2005). These results indicate that photosynthesis, which produces oxygen, significantly contributes to the energy status of developing seeds. Interestingly, in an independent study, overexpression of hemoglobin 2 in *Arabidopsis* seeds improved the energy status of seeds under low oxygen conditions and led to an increase in the fatty acid content of developing and mature seeds, further emphasizing the importance of oxygen availability during seeds development (Vigeolas et al., 2011). Photosynthesis could also be important in relatively large seeds, such as soybean seeds, whose inner cell layers receive significantly lower oxygen levels that the outer seed tissues. Borisjuk et al. (2005) demonstrated that developing dicot seeds with green embryos display a reduction in photosynthetic activity during development, starting from the interior of the embryo. This indicates the contribution of this photosynthetic activity to seed energy status.

Photosynthesis encompasses two different reactions, namely: (i) the light reactions that lead to reduction of NADP to NADPH and create a proton gradient across the chloroplast membrane that is used for ATP synthesis; and (ii) the light-independent reactions in which RuBisCO fixes CO_2_ from the atmosphere in a NADPH-requiring process, and the Calvin–Benson cycle produces sugars (Buchanan et al., 2000). Interestingly, developing oilseed rape (*Brassica napus* L) embryos possess active photosynthesis, and RuBisCO acts in a special metabolic context without the Calvin–Benson cycle in order to improve the efficiency of carbon utilization during the synthesis of oil storage (Schwender et al., 2004a). This special pathway generates 20% more acetyl-CoA, a precursor for fatty acid biosynthesis, in comparison to glycolysis and saves 40% of the carbon that would otherwise be lost as CO_2_.

## CONTRIBUTION OF AMINO ACID METABOLISM TO THE ENERGY STATUS OF DEVELOPING SEEDS

Amino acids are constituents of proteins and hence essential components for the life of all organisms. Yet, in response to specific developmental and stress-associated conditions, amino acids also serve as energy donors through their catabolism in the TCA cycle (Araujo et al., 2010; Angelovici et al., 2011; Kirma et al., 2012). In vegetative tissues, photosynthesis is the major source of energy during daytime, and thus during daytime amino acids are used for the synthesis of proteins and, as precursors of multiple secondary metabolites (Less and Galili, 2008). Yet, during night-time or in response to stresses that cause major energy shortages due to the lack of or reduction in photosynthesis, amino acids also serve as important energy sources through their catabolism via the TCA cycle (Araujo et al., 2011). Therefore, the contributions of amino acid catabolism to the energy status requirements of developing seeds appears even more critical than in vegetative tissues, due to the limits of oxygen diffusion.

## CONTRIBUTION OF Lys SYNTHESIS AND CATABOLISM IN THE TCA CYCLE TO SEED ENERGY STATUS

Insight into the potential importance of Lys catabolism to the energy status of developing seeds was obtained in research aiming to enhance the nutritional quality of seeds by increasing Lys accumulation (Zhu and Galili, 2003). Lys is an essential amino acid that serves a vital role in human food and livestock feed, since humans and some livestock (such as chicken and pigs, i.e., mono-gastric livestock) are unable to synthesize Lys (Kirma et al., 2012). The synthesis of Lys in plants (**Figure 1**) is subject to post-translational regulation, where increasing Lys feedback inhibits the activity of dihydrodipicolinate synthase (DHDPS), which catalyzies the first committed step in the Lys biosynthetic branch of the Asp-family pathway. Plants possess two DHDPS isozymes (DHDPS1 and DHDPS2), with DHDPS2 accounting for the majority of the total DHDPS activity (Jones-Held et al., 2012). Because Lys is an essential amino acid, an attempt was made to increase Lys in seeds of the model plant *Arabidopsis*, using a recombinant gene encoding in a seed-specific manner a mutant bacterial DHDPS2 enzyme insensitive to Lys feedback inhibition (Zhu and Galili, 2003). This approach yielded only a relatively mild increase in seed Lys, leading to the hypothesis that this amino acid is not an end product metabolite, but rather might be catabolized in the TCA cycle to generate energy. To test this hypothesis, the same bacterial feedback-insensitive DHDPS was expressed in seeds of a transgenic *Arabidopsis* genotype that lacks the capacity for Lys catabolism due to a knockout mutation in the gene encoding the bi-functional enzyme lysine-ketoglutarate reductase/saccharopine dehydrogenase (LKR/SDH), which contains the first two enzymes of Lys catabolism linked as a single polypeptide. In this combined genotype, seed Lys content was nearly 1000-fold higher than wild type (WT), indicating that developing seeds have a strong flux of Lys synthesis as well as catabolism into the TCA cycle (Zhu and Galili, 2003).

Developing seeds possessing significantly enhanced Lys levels due to increased Lys synthesis and suppression of Lys catabolism also exhibited notable differences in their transcriptomes and primary metabolomes, indicating that Lys metabolism (synthesis and catabolism) is well connected to the TCA cycle (Angelovici et al., 2009, 2011). For example, compared to WT, the levels of the TCA cycle metabolites fumarate, citrate, and ketoglutarate are lower in the genotype possessing enhanced Lys synthesis and a block of Lys catabolism, compared to the wild-type, genotype (Angelovici et al., 2009, 2011). Lys catabolism into the TCA cycle is apparently used to generate energy essential for seed development. As shown in **Figure 1**, Lys is not the only amino acid of the Asp-family pathway that feeds into the TCA cycle. A second branch of the Asp-family pathway leads to the synthesis of Thr and Met, which are further metabolized into Ile, which also feeds into the TCA cycle and serves as a major substrate for energy generation (Joshi et al., 2006; Kochevenko and Fernie, 2011).

In the developing seed, the upstream substrate that feeds the Asp-family pathway on route to the TCA cycle is Asn. Asn is transported from vegetative tissues to developing seeds where it is converted by asparaginase into Asp, the starting point metabolite of the Asp-family pathway (**Figure 1**; Credali et al., 2013). Indeed, the asparaginase level is generally stimulated during early seed development (Dickson et al., 1992; Credali et al., 2013).

## AMINO ACID CATABOLISM FACILITATES RESPIRATION UNDER STRESS CONDITIONS

Under normal conditions, respiration depends on the oxidation of carbohydrates. However, during situations in which carbohydrate supply is limited, the plant cell can modify its metabolism to utilize alternative respiratory substrates. Among these substrates are proteins. Protein degradation is a highly regulated process, involving a multitude of cellular reactions, such as ubiquitinylation and degradation *via* the proteasome, the autophagy machinery and nutrient sensing by the TOR pathway. The product of protein degradation, free amino acids, can be further catabolize to generate energy (ATP; Zhu and Galili, 2003; Joshi et al., 2006; Araujo et al., 2011; Kochevenko and Fernie, 2011). Indeed, it has been shown that under abiotic stress there is increased transcription of amino acid catabolic genes (Less and Galili, 2008).

**FIGURE 2:**
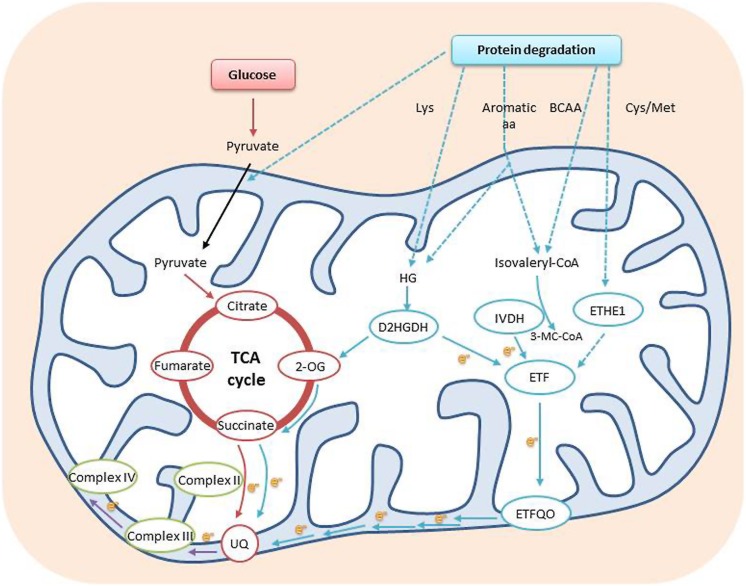
**Amino acids can be used as electron donors to the mitochondrial electron transport chain.** Protein degradation yields a range of amino acids that are further metabolized either into isovaleryl-CoA or HG. Isovaleryl-CoA can be produced by catabolism of the branched chain and aromatic amino acids and by both phytol and Lys degradation, whereas HG can be produced by aromatic amino acid degradation or from the Lys derivative L-pipecolate. The electrons generated are transferred to the respiratory chain through to the ubiquinol pool via an ETF/ETFQO system. Possible involvement of sulfur containing amino acids has also been implicated by the phenotype of ETHE1 knockdown plants. Some amino acids can facilitate energy production *via* the TCA cycle, either by conversion to pyruvic acid or acetyl-CoA or by direct conversion to TCA cycle intermediates, such as 2-OG, and direct electron supply to the ubiquinone pool of the mitochondrial electron transport chain in plants. Dotted arrows represent possible transport processes and multi enzymatic reactions. Abbreviations: BCAA, branched chain amino acids; D2HGDH, 2-D-hydroxyglutarate dehydrogenase; e–, electron; ETF, electron transfer flavoprotein; ETFQO, ETF:ubiquinone oxidoreductase; ETHE1, ethylmalonic encephalopathy protein1; HG, hydroxyglutarate; IVDH, isovaleryl-CoA dehydrogenase; 3-MC-CoA, 3-methylcrotonyl-CoA; 2-OG, 2-oxoglutarate; TCA cycle, tricarboxylic acid cycle; UQ, ubiquinone (Adapted from Araujo et al., 2011; Krüssel et al., 2014).

In mammals, the mitochondrial protein, electron-transfer flavoprotein:ubiquinone oxireductase (ETFQO), accepts electrons from electron transfer flavoprotein (ETF) to reduce ubiquinone. ETF serves as an obligatory electron acceptor for nine mitochondrial flavoprotein dehydrogenases. The ETF/ETFQO system facilitates electron transfer from these flavoprotein dehydrogenases to the main respiratory chain (Ishizaki et al., 2005, 2006). Homologs of ETF and ETFQO have been characterized in plants, as well as two flavoprotein dehydrogenases: isovaleryl-CoA dehydrogenase (IVDH) and 2-D-hydroxyglutarate dehydrogenase (D2HGDH; Ishizaki et al., 2005, 2006; Araujo et al., 2010). T-DNA insertion mutants of each of these genes display increased sensitivity to prolonged darkness, a condition that leads to carbon starvation. In addition, metabolic analysis of these mutants under prolonged darkness revealed an accumulation of several amino acids and an intermediate metabolite of Leu catabolism in comparison to WT. These results suggest a role for amino acid catabolism in supplying respiratory intermediates during carbon starvation (**Figure 2**; Ishizaki et al., 2005, 2006). It has been demonstrated that IVDH is apparently predominantly responsible for the transport of electrons from breakdown products of phytol and branched-chain amino acids (BCAA: Ile, Leu, and Val), while D2HGDH is responsible for the transport of electrons from breakdown products of Lys (**Figure 2**). This last finding is interesting, as it demonstrates the ability of Lys degradation products to contribute electrons directly to the mitochondrial electron transport chain, as well as supply intermediates to the TCA cycle (Araujo et al., 2010).

Although a direct role for the ETF/ETFQO system in seed development has not yet been demonstrated, considerable experimental evidence has accumulated to support this hypothesis. First, the existence of hypoxic conditions and lack of photosynthesis in some seeds point to the need for alternative electron donors in seeds for the mitochondrial electron transport chain. Secondly, analysis of a mutant with high levels of 12 out of the 20 amino acids in seeds was shown to be IVDH defective, further strengthening the role of this enzyme in the regulation of free amino acid homeostasis during seed development (Gu et al., 2010). Thirdly, a recent publication highlighted the role of BCAT2, a BCAA catabolic enzyme, in establishing the variation of BCAA levels in different *Arabidopsis* accessions (Angelovici et al., 2013). BCAT2 was shown to localize in the mitochondria, and its expression was shown to increase during seed development, suggesting the involvement of BCAA catabolism in seed development, possibly via the ETF/ETFQO system (Angelovici et al., 2013). Additional evidence suggesting the involvement of the ETF/ETFQO system in seed development was obtained using genetic manipulation of amino acid delivery to pea (*Pisum sativum*) embryos. When an increase of amino acid delivery was induced, transcripts of the ETF complex were most highly induced (Weigelt et al., 2008). It is important to note that *etf* and *etfqo* mutants display lower seed set and shorter siliques when grown under long day conditions (Ishizaki et al., 2005, 2006). This phenotype is attributed to the maternal tissue, as demonstrated by reciprocal crosses of etfqo mutants and WT plants (Ishizaki et al., 2005). Another finding strengthening the role of the ETF/ETFQO system in seed development is a recent publication describing ETHE1, a mitochondrial sulfur dioxygenase. ETHE1 is a matrix protein that has been shown to be involved in the detoxification of sulfur compounds derived from sulfur-containing amino acids (Cys and Met). A complete knockout of ETHE1 is embryo lethal and ETHE1 knockdown plants are viable, but present a delay in seed development. Surprisingly, ETHE1 knockdown plants display higher sensitivity to carbon starvation, reminiscent of the ETF/ETFQO system T-DNA mutants (**Figure 2**) and have been shown to accumulate BCAA under these conditions. These data suggest a possible role for ETHE1 in the ETF/ETFQO system as well as hinting at the importance of the enzyme, *per se*, during seed development (Krüssel et al., 2014).

*In summary,* in this review we discussed a number of studies that have enhanced our understanding of the parallel roles of photosynthesis and amino acid-derived respiratory catabolic processes in meeting the demands for normal energy status during seed development. In respect to amino acids, we focused on Lys degradation and highlighted the involvement of the ETF pathway. Technical advances, including improved understanding of sub-cellular compartmentization of metabolism (Sweetlove and Fernie, 2013), alongside mathematical approaches to understand whole plant physiology as well as metabolite-metabolite associations (Toubiana et al., 2012; Grafahrend-Belau et al., 2013) are now available. These will greatly enhance our understanding of these enzymatic processes. Furthermore, metabolite imaging techniques, such as genetically encoded metabolite sensors (Okumoto et al., 2012), nuclear magnetic imaging (Borisjuk et al., 2013), and flux profiling techniques (Schwender et al., 2004b), alongside miniaturization of respiration measurements (Sew et al., 2013), will additionally enhance our understanding of the shifts in plant energy metabolism that occur during the process of seed development. Our potential to metabolically engineer seeds in a highly tailored manner will be radically improved once such information is available and integrated with our current knowledge.

## Conflict of Interest Statement

The authors declare that the research was conducted in the absence of any commercial or financial relationships that could be construed as a potential conflict of interest.
